# A Pilot Study Investigating Clinical and Functional Outcomes of Novel Double-Coil rPMS in Knee Osteoarthritis

**DOI:** 10.3390/biomedicines14030722

**Published:** 2026-03-20

**Authors:** Roman Bednár, Martina Flašková, Nicole Fejková

**Affiliations:** Department of Physical Medicine, Balneology, and Rehabilitation, F. D. Roosevelt Faculty Hospital with Polyclinic, 975 17 Banská Bystrica, Slovakia

**Keywords:** repetitive peripheral magnetic stimulation, knee joint, osteoarthritis, double-coil rPMS configuration

## Abstract

**Background:** Knee osteoarthritis (KOA) is one of the leading causes of chronic pain and long-term disability worldwide. Despite its high prevalence, KOA remains underrepresented in repetitive peripheral magnetic stimulation (rPMS) research. While total knee arthroplasty remains the definitive treatment, there is a growing need for non-invasive approaches to reduce symptoms in patients seeking conservative alternatives or awaiting surgery. **Methods:** Thirty patients with KOA underwent a non-invasive treatment program consisting of eight sessions of double-coil repetitive peripheral magnetic stimulation (rPMS) over three weeks. Outcome measures included pain intensity assessed by the Visual Analog Scale (VAS), functional ability evaluated by the Western Ontario and McMaster Universities Osteoarthritis Index (WOMAC) and the Timed Up and Go test (TUG), and joint mobility measured as knee flexion and extension. Clinical relevance was evaluated using the Minimal Clinically Important Difference (MCID), and subgroup analyses were performed according to Kellgren-Lawrence (KL) grade. **Results:** Double-coil rPMS was associated with statistically and clinically significant improvements in all outcomes. MCID responder rates exceeded 80% for VAS and TUG, exceeded 70% for WOMAC, and approached 50% for joint mobility outcomes. Subgroup analysis indicated that patients with lower KL grades experienced greater pain reduction, whereas those with higher grades showed greater functional gains. **Conclusions:** Double-coil rPMS provided preliminary evidence of potential clinical benefit as a non-invasive approach in patients with KOA. Given the single-arm pilot design, the findings should be interpreted cautiously and require confirmation in adequately powered randomized controlled trials with longer follow-up.

## 1. Introduction

Osteoarthritis (OA) is one of the leading causes of chronic pain and long-term disability among adults [1]. Although this degenerative disease can affect various joints, up to 80% of all cases involve the knee joint, making knee osteoarthritis (KOA) the most prevalent form [2]. Traditionally, osteoarthritis has been regarded as a progressive wear-and-tear process of the articular cartilage. However, accumulating evidence suggests that OA is an inflammatory disease of the entire synovial joint, involving not only mechanical degeneration of articular cartilage but also concurrent structural and functional changes in multiple joint tissues, including the synovium, meniscus, periarticular ligaments, subchondral bone, and the infrapatellar fat pad, which has been recognized as an active source of inflammatory mediators contributing to pain and disease progression [3]. The condition is strongly associated with advanced age and obesity; approximately 73% of individuals with OA are older than 55 years and about 31% are classified as obese [4,5]. Moreover, women are disproportionately affected, representing approximately 60% of all OA patients [4].

In addition to obesity, sex and advanced age, several other risk factors have been identified for the development of KOA, including previous joint injury, physically demanding occupations, high-level sports participation, surgical procedures such as meniscectomy following trauma, joint anatomical variations, and muscle weakness [1,6]. KOA, as a degenerative disorder of the knee joint, results from mechanical stress and progressive cartilage degradation [7]. These degenerative changes are often associated with prolonged standing, repetitive knee bending, or heavy lifting [6,7]. The main clinical manifestations of KOA include pain, swelling, and stiffness, which substantially impair patients’ functional capacity [7]. However, a considerable proportion of individuals remain asymptomatic, with estimates suggesting that 4–14% of patients under the age of 40 and 19–43% of those over 40 years exhibit radiographic signs of KOA without clinical symptoms [8].

The most common diagnostic tools for KOA include X-ray, magnetic resonance imaging (MRI), and arthroscopy. To classify the severity of knee OA, the Kellgren-Lawrence (KL) grading system was developed, which assigns patients to a five-point scale based on radiographic findings such as joint space narrowing, osteophyte formation, subchondral sclerosis, and bone deformity [9,10]. A normal radiographic appearance corresponds to grade 0, while severe KOA is defined as grade 4 [9]. Although no treatment is currently available to reverse the progression of osteoarthritis, various pharmacological and non-pharmacological interventions aim to alleviate symptoms and improve function [11]. Exercise-based therapy has been shown to improve pain and functional outcomes and may modulate oxidative stress and inflammatory pathways involved in KOA progression [12]. In cases of advanced KOA associated with severe disability, surgical intervention in the form of total knee arthroplasty is often required [10]. Despite clinical guidelines recommending conservative non-pharmacological interventions as the first-line management for KOA, only 40–65% of patients receive appropriate non-surgical care, while pharmacological and surgical strategies continue to dominate overall treatment practice [13].

In addition to dietary management, exercise, and manual therapy, several other non-invasive conventional methods have demonstrated effectiveness in alleviating the symptoms of KOA, including cryotherapy, electrical stimulation, extracorporeal shock wave therapy, laser therapy, ultrasound, and magnetic field therapy [13]. Conventional low-intensity magnetic therapy devices typically generate magnetic field strengths below 50 mT, whereas modern high-intensity magnetic stimulation systems are capable of producing fields of up to 3 T. Recent technological advances have led to the development of high-performance peripheral magnetic stimulation systems employing multidirectional or double-coil configurations, allowing for deeper and more homogeneous stimulation of musculoskeletal tissues [14,15].

Current clinical practice increasingly favors low-frequency electromagnetic fields, which induce electrical currents in exposed tissues and can effectively stimulate neuromuscular structures [16]. Repetitive peripheral magnetic stimulation (rPMS) is widely used in the treatment of musculoskeletal pain and is based on the principle of electromagnetic induction, whereby a time-varying magnetic field passes through nerve and muscle tissue, generating electric currents without direct skin contact [17]. rPMS penetrates deeper conductive structures and allows relatively painless stimulation of peripheral neuromuscular tissues [18,19].

Through the action of induced electrical currents, rPMS may reduce the transmission efficiency of nociceptive signals from the periphery to the central nervous system, particularly under inflammatory conditions. The use of low-frequency stimulation in the range of 10–20 Hz has been shown to beneficially affect periarticular soft tissues by increasing tissue elasticity, reducing muscular hypertonus, and decreasing edema through activation of the microvascular pump. Additional biophysical effects include improved dispersion of interstitial fluids and enhanced local perfusion, metabolism, and tissue trophicity. For degenerative musculoskeletal conditions requiring muscle activation, repeated contractions, and support of regenerative and reparative processes, high-intensity magnetic stimulation may represent a promising therapeutic modality [20].

Through the effect of intense muscle activation, rPMS has been shown to reduce spasticity and improve functional disability across various neurological conditions [21,22]. However, evidence regarding its application in KOA remains limited. To date, only a single study employing a multi-session treatment protocol has specifically investigated the effects of rPMS in patients with KOA, involving a small sample size (10 participants per group) and a relatively demanding treatment protocol consisting of 20 sessions over one month [23]. Furthermore, existing systems rely on a single-coil setup, in which magnetic field intensity decreases with tissue depth, potentially limiting effective stimulation of deeper joint structures [24]. In addition, single-coil stimulation is typically applied from only one side of the joint, which restricts the spatial distribution of the magnetic field and may result in asymmetric tissue activation. Consequently, simultaneous targeting of multiple joint compartments or opposing anatomical structures is not feasible with conventional single-coil configurations.

**Figure 1:**
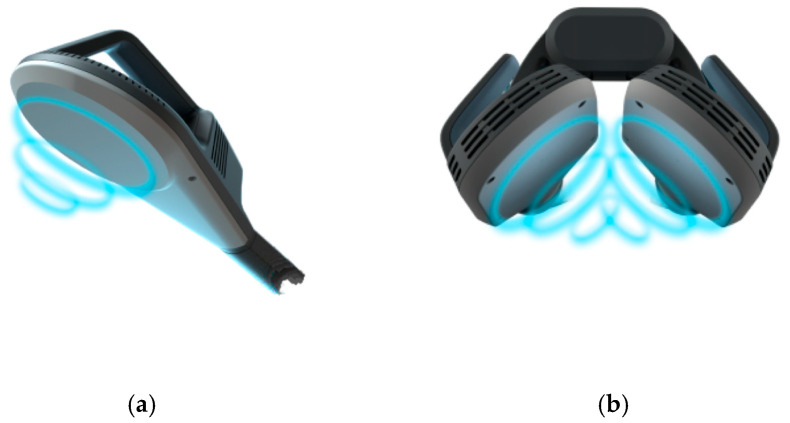
Schematic comparison of magnetic field distribution in single-coil (**a**) and double-coil (**b**) rPMS configurations. Image reproduced with permission from BTL Industries.

The limited clinical evidence supporting rPMS in the treatment of such a prevalent condition as KOA may be partly attributed to the currently inefficient distribution of the magnetic field, which is typically applied using a single coil positioned perpendicularly above the target area. The intensity of the magnetic field decreases with tissue depth, and stimulation of deeper joint structures may therefore not always be sufficiently effective [24]. A potential breakthrough in the treatment of large joints could be achieved through the use of a novel double-coil setup, which allows adjustment of the relative angle between two coils and their placement on opposite sides of the treated joint. Theoretical and simulation-based analyses suggest that such a setup may generate a stronger and more homogeneous magnetic field at greater depths compared with conventional single-coil stimulation [22,23]. A schematic comparison of magnetic field distribution in single- and double-coil configurations is presented in Figure 1.

However, this proposed advantage has not yet been validated in clinical KOA populations. Therefore a clear research gap exists regarding the clinical effectiveness of double-coil rPMS in patients with knee osteoarthritis, particularly across different stages of radiographic severity.

The aim of this study is to investigate the effects of a novel double-coil rPMS on pain and functional disability in patients with different grades of KOA. Additionally, the study aimed to evaluate the influence of KOA grade on the percentage changes in individual outcome measures following treatment.

## 2. Materials and Methods

### 2.1. Study Design

This study was designed as a prospective, single-arm pilot clinical trial evaluating the effects of a novel double-coil rPMS protocol in patients with KOA. The research was conducted as part of routine clinical care at the F. D. Roosevelt Faculty Hospital with Polyclinic in Banská Bystrica, Slovakia. Ethical approval was obtained from the Institutional Ethics Committee of F.D. Roosevelt University Hospital (Banská Bystrica, Slovakia) on 14 October 2025 (approval number 17/2025) prior to patient recruitment, and the study was conducted in accordance with the ethical principles of the Declaration of Helsinki. All participants were informed about the purpose, potential benefits, and risks of the intervention, as well as the possibility of data publication, and provided written informed consent before treatment initiation.

Given the exploratory and feasibility-oriented nature of this pilot study, a control or sham group was not included. The primary objective was to assess safety, tolerability, and preliminary clinical response of the double-coil configuration under routine clinical conditions. The results are therefore intended to be hypothesis-generating and to inform the design of future randomized controlled trials.

### 2.2. Participants

Patients diagnosed with knee osteoarthritis (KL grades II–IV) were eligible for inclusion in the study. No age restriction was applied. Only patients with active knee flexion of at least 90° and the ability to achieve full weight-bearing during ambulation without assistive devices were included. Stable chronic symptoms were defined as persistent knee pain for at least three months without acute exacerbation requiring treatment modification within the preceding four weeks. Osteoarthritis of other joints was permitted if present in a compensated and clinically stable stage. Participants were required to be at least one month after intra-articular knee injection and at least six months after knee arthroscopy. Participants were instructed to maintain their usual level of physical activity and stable analgesic medication throughout the study period. Any medication changes were required to be reported.

Patients were excluded if they were pregnant or had any implanted metallic or electronic devices, such as pacemakers, defibrillators, neurostimulators, or metal-containing intrauterine devices. Additional exclusion criteria included the presence of active drug infusion systems, a history of seizures or epilepsy, active malignancy, systemic infection, or open skin lesions in the treatment area. Individuals with severe cardiovascular, pulmonary, or renal disease, febrile conditions, or any neurological or musculoskeletal disorder unrelated to KOA that might affect functional outcomes were also excluded.

### 2.3. Intervention Protocol

**Figure 2:**
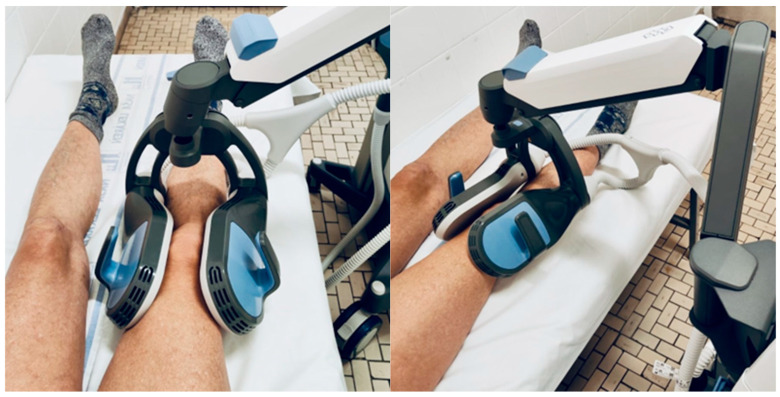
Setup of the double-coil repetitive peripheral magnetic stimulation (rPMS) during treatment.

Participants received treatment in a supine position. The double-coil applicator was positioned laterally and medially around the knee joint, forming a 90° angle between the coils (Figure 2). Treatment was delivered using a commercially available high-intensity magnetic stimulation device (BTL SIS DUO, BTL Industries, Ltd., Prague, Czech Republic), equipped with a dual-coil applicator capable of generating a maximal magnetic flux density of 2.4 T at each coil surface. The device complies with IEC 60601-1 safety standards and is classified as a Type BF medical device [25]. Treatment sessions were performed by multiple physicians specialized in Physical and Rehabilitation Medicine (PRM), who were trained in the application of the double-coil rPMS protocol prior to study initiation. All PRM physicians followed a standardized treatment procedure, including predefined applicator positioning and stimulation parameters, in order to minimize inter-operator variability. Stimulation intensity was recorded after each session for every patient. During subsequent sessions, the same intensity was applied or adjusted, with any increase limited to a maximum of 5% based on individual tolerance and always agreed upon with the patient. This approach ensured both dose consistency and patient safety throughout the treatment course. The inter-coil angle was adjusted based on the location of the patient’s primary pain within the knee, enabling individualized and anatomically targeted stimulation.

A pre-programmed protocol specifically developed for chronic pain conditions such as osteoarthritis was used. Across the protocol, stimulation was delivered using a combination of constant, alternating, and sinusoidal modulation patterns, with frequency varied within a predefined therapeutic range of approximately 5–50 Hz, incorporating both low-frequency neuromodulatory components and higher-frequency neuromuscular activation phases. Amplitude parameters varied within predefined therapeutic ranges. The total treatment duration was 13 min, consisting of six sequential phases:

1. Configuration phase: device setup and positioning;
2. Preparation phase: gradual increase in stimulation intensity to achieve optimal neuromuscular activation;
3. Effective Pain Control phase: frequency modulation with low-frequency stimulation aimed at reducing pain intensity through the activation of endogenous opioid pathways, consistent with the endorphin theory of analgesia [26];
4. Effective Pain Control phase: stimulation based on the same analgesic principle as Phase 3;
5. Enhanced Healing and Blood Flow phase: amplitude modulation designed to improve local circulation and facilitate tissue repair [27];
6. Calming phase: gradual reduction in stimulation intensity to ensure a comfortable end of treatment and to allow device cooling while maintaining stable output performance;

Stimulation intensity was individually adjusted according to patient tolerance and modified as necessary during each session. Each participant underwent eight treatment sessions over a three-week treatment course.

### 2.4. Outcome Measures

Four validated clinical outcome measures were used to assess treatment effectiveness: pain intensity, overall knee function, joint mobility, and functional mobility. Assessments were performed at baseline and after completion of the intervention period, with pain additionally assessed halfway through the treatment course. To evaluate clinical relevance, the proportion of patients who achieved the minimal clinically important difference (MCID) was also determined for each measure.

Pain intensity was evaluated using the Visual Analog Scale (VAS), a 10 cm horizontal line anchored by “no pain” and “worst pain imaginable.” Participants marked their perceived pain level on the line, and the score was recorded in centimeters from the left end (0–10 scale) [28]. A decrease in VAS score indicates a reduction in pain intensity and therefore clinical improvement. The MCID value for VAS was set at 2.0 cm, based on available evidence for KOA [29].

Overall disease status was assessed using the Western Ontario and McMaster Universities Osteoarthritis Index (WOMAC), which evaluates pain, stiffness, and physical function [30]. The WOMAC questionnaire was administered in its normalized 0–100 version, where higher scores indicate better function and less pain. Accordingly, an increase of ≥6 points was considered the MCID [31].

Joint mobility was measured as the range of motion (ROM) of the affected knee using a standard goniometer. Flexion and extension were recorded in degrees under consistent conditions by the same examiner [32]. An increase in knee flexion reflects improved joint mobility, whereas a decrease in the extension deficit (i.e., a smaller measured value) indicates better knee function. A change of ≥5° in flexion or ≤−5° in extension was considered clinically meaningful in KOA rehabilitation [33].

Functional mobility was assessed using the Timed Up and Go (TUG) test. Participants were instructed to rise from a chair, walk three meters, turn around, return, and sit down. The same chair was used for all tests, maintaining identical characteristics such as seat height, presence of armrests, and back support [34]. A decrease in TUG time indicates improved functional mobility. The MCID for TUG performance in individuals with KOA has been reported as at least 0.8 s [33].

### 2.5. Statistical Analysis

Statistical analyses were performed using IBM SPSS Statistics (Version 29.0) [35]. Data distribution was assessed for normality using the Shapiro–Wilk test. Variables with a normal distribution were presented as mean ± standard deviation (SD), and within-group differences were evaluated using the paired *t*-test. If the normality assumption was violated, data were expressed as median (interquartile range), and changes were analyzed using the non-parametric Wilcoxon signed-rank test. For VAS, measured at three time points, repeated measures ANOVA was used to assess the effect of time, with effect size expressed as partial eta squared (η^2^p).

Given the pilot nature of the study and the limited sample size, the magnitude of improvement was further explored through complementary statistical indicators. For each variable, 95% confidence intervals were calculated for the mean or median change, and effect sizes were computed to quantify the clinical relevance of the observed differences. For normally distributed data, effect size was expressed as Cohen’s d, whereas for non-normally distributed data, rank-biserial correlation (r) was used. According to conventional benchmarks, values of 0.2, 0.5, and 0.8 for d, or 0.1, 0.3, and 0.5 for r, were interpreted as small, medium, and large effects, respectively.

Additionally, MCID responder rates were calculated for each outcome. Patients whose baseline values did not allow for a potential improvement equal to or exceeding the predefined MCID were excluded from the MCID responder analysis to minimize ceiling and floor effects.

To further explore whether disease severity independently influenced treatment response, an exploratory multivariable linear regression analysis was performed. Change in VAS score (Pre–Post) was used as the dependent variable, with KL grade, age, and BMI entered as independent predictors. This analysis was conducted to assess potential confounding effects while acknowledging the limited statistical power associated with the small sample size.

In an additional analysis aimed at evaluating the effect of KL grade on treatment outcomes, percentage improvements in each outcome measure were calculated separately for patient subgroups with KL grade < 3 and KL grade ≥ 3. Between-group differences in the magnitude of change were analyzed using the Mann–Whitney U test. A significance level of *p* < 0.05 was considered statistically significant for all analyses.

As this was an exploratory pilot study designed to evaluate the feasibility and preliminary efficacy of a novel intervention, no formal sample size calculation was performed. The primary objective of this study was not to test a hypothesis of superiority but to obtain initial estimates of variability and effect size to inform the design of future randomized controlled trials. The sample size was determined pragmatically based on the expected recruitment rate within the study period and was consistent with published recommendations for pilot clinical trials, which suggest 12–35 participants per treatment arm to estimate variance and effect size for future studies [36]. Therefore, the present study should be interpreted as hypothesis-generating rather than confirmatory.

The multivariable analysis was exploratory in nature and should be interpreted with caution given the limited statistical power.

## 3. Results

Of the 33 enrolled patients, 30 (23 women and 7 men) completed the full treatment program. One participant withdrew due to unexpected travel abroad, and two discontinued participation because of unrelated medical conditions. Additionally, one participant reported a single dose of non-steroidal anti-inflammatory medication at the beginning of therapy; no other medication changes were recorded. No adverse events were reported during the study period.

The applied stimulation intensity averaged 26.1% of the device output, with values ranging from 3% to 85%, and was adjusted individually according to patient tolerance. The mean age of the cohort was 61.37 ± 8.22 years. The average body mass index (BMI) was 28.33 ± 5.59 kg/m^2^, indicating a predominance of overweight to obese individuals in the treated group. A total of 14 patients had KL grade < 3, and 16 patients had KL grade ≥ 3. The mean overall KOA grade was 2.65 ± 0.50.

**Figure 3:**
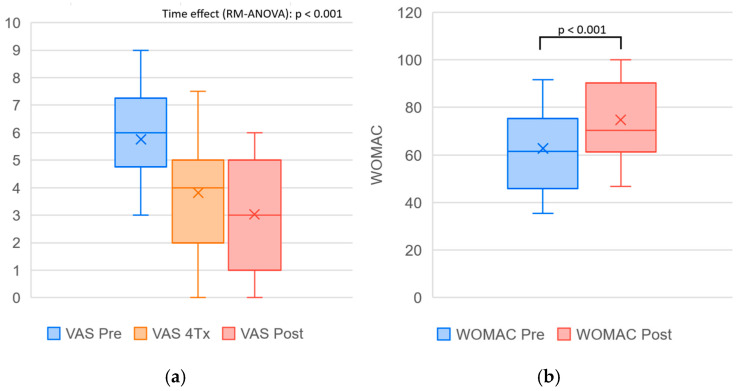
Boxplots illustrating changes in (**a**) pain intensity assessed by the Visual Analog Scale (VAS) and (**b**) functional status assessed by the Western Ontario and McMaster Universities Osteoarthritis Index (WOMAC) across the treatment course. VAS was assessed at baseline (Pre), after four treatment sessions (4Tx), and after completion of treatment (Post); WOMAC was assessed at Pre and Post. VAS differences across three time points were analyzed using repeated measures ANOVA, and WOMAC changes (Pre vs. Post) were evaluated using a paired *t*-test. Each box represents the interquartile range (IQR), the horizontal line indicates the median, and “×” marks the mean value.

**Figure 4:**
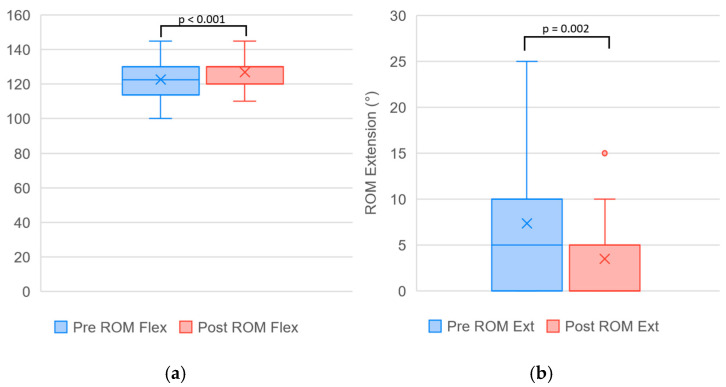
Boxplots illustrating changes in (**a**) knee flexion and (**b**) knee extension range of motion (ROM) across the treatment course (Pre vs. Post). Within-group differences were analyzed using the Wilcoxon signed-rank test. Each box represents the interquartile range (IQR), the horizontal line indicates the median, and “×” marks the mean value. Whiskers denote minimum and maximum values excluding outliers (circles).

**Figure 5:**
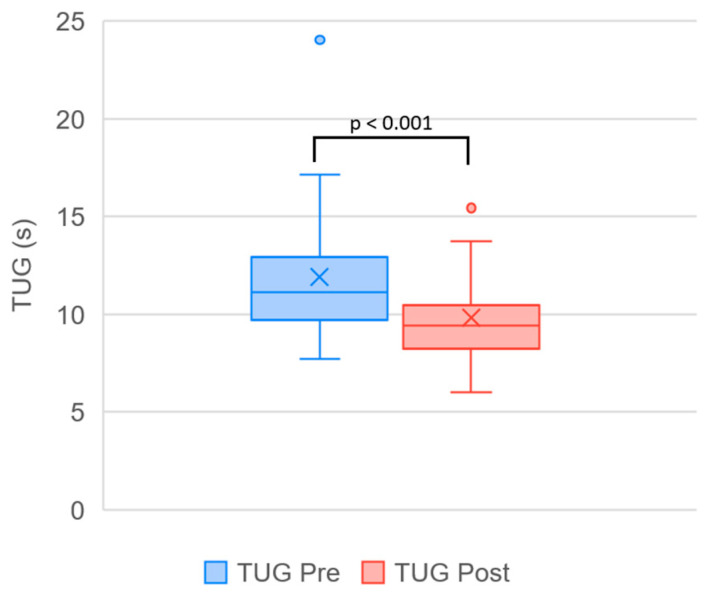
Boxplot illustrating changes in Timed Up and Go (TUG) performance across the treatment course (Pre vs. Post). Within-group differences were analyzed using the Wilcoxon signed-rank test. Each box represents the interquartile range (IQR), the horizontal line indicates the median, and “×” marks the mean value. Whiskers denote minimum and maximum values excluding outliers (circles).

A summary of outcome measures obtained before and after completion of the treatment program, together with the results of the statistical analysis, is presented in Table 1. Changes observed over the course of the program reached statistical significance for all evaluated parameters and represent treatment-associated changes within this pilot cohort. The effect of time on VAS was large (η^2^p = 0.605), and all other outcomes demonstrated large effect sizes. A visual comparison of the data distribution is provided in Figure 3, Figure 4 and Figure 5.

**Figure 6:**
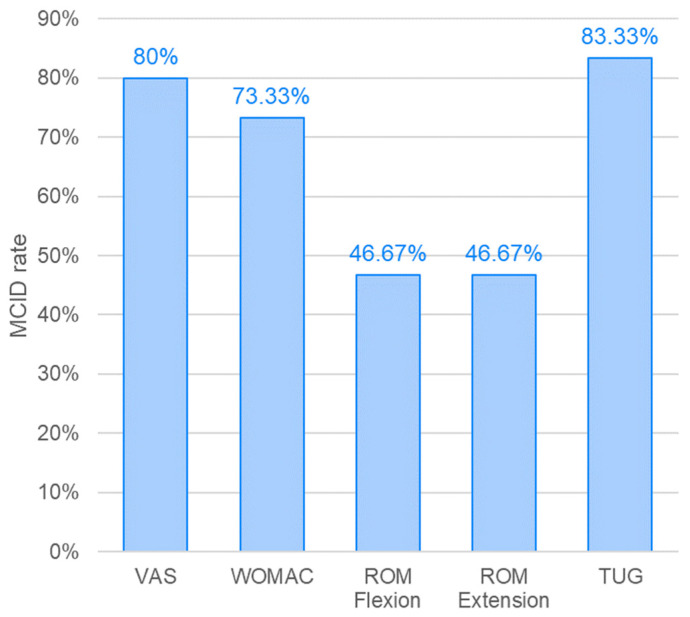
Proportion of patients achieving the minimal clinically important difference (MCID) for each outcome measure. MCID thresholds were defined as ≥2 cm for the Visual Analog Scale (VAS), ≥6 points for the Western Ontario and McMaster Universities Osteoarthritis Index (WOMAC), ≥5° for knee flexion range of motion (ROM), ≤−5° for knee extension ROM, and ≥0.8 s for the Timed Up and Go (TUG) test. Bars represent the percentage of patients exceeding the predefined MCID threshold, indicating clinically meaningful improvement.

The MCID responder rate for knee range of motion, including both flexion and extension, reached approximately 47%, whereas for the other outcome measures it was considerably higher, ranging between 70% and 80%. The percentage of patients achieving the MCID threshold for each parameter is graphically presented in Figure 6.

An exploratory multivariable linear regression analysis was performed with change in VAS score (Pre–Post) as the dependent variable and KL grade, age, and BMI as independent predictors. The overall model was not statistically significant (F(3.26) = 0.92, *p* = 0.446, R^2^ = 0.096), and none of the included variables were identified as independent predictors of VAS improvement.

**Figure 7:**
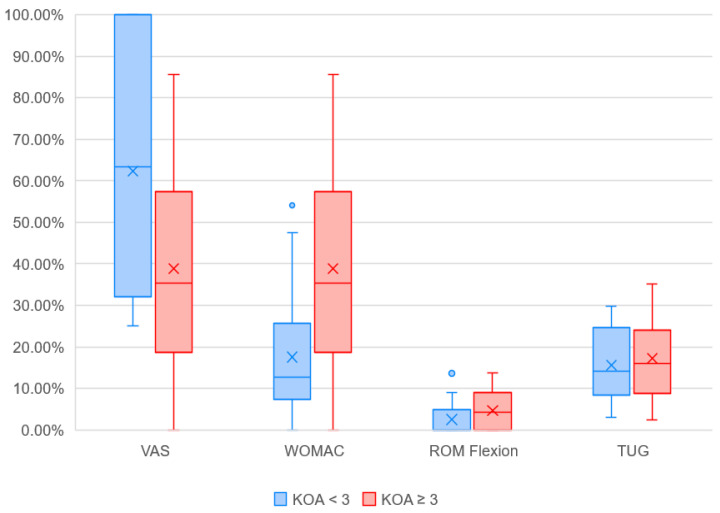
Between-group comparison of treatment-induced percentage improvements (Δ%) in the Visual Analog Scale (VAS), Western Ontario and McMaster Universities Osteoarthritis Index (WOMAC), knee flexion range of motion (ROM flexion), and Timed Up and Go (TUG) performance between patients with knee osteoarthritis classified as Kellgren–Lawrence (KL) grade < 3 and ≥3. Boxes represent interquartile ranges (IQR), horizontal lines indicate medians, and “×” marks mean values. Whiskers denote minimum and maximum values excluding outliers (circles). Positive values indicate improvement compared to baseline (i.e., reduction in pain or time, or increase in function or mobility). Knee extension ROM was analyzed separately, as it was expressed as absolute change in degrees (°) rather than percentage change.

Changes in outcome measures for patient subgroups according to KL grade of KOA are summarized in Table 2 and visually illustrated in Figure 7. While patients with lower KL grades reported greater improvement in pain reduction, those with KL grade ≥ 3 demonstrated larger benefits in WOMAC scores and range of motion. Although the visual differences between the subgroups appear substantial, statistical significance was reached only for changes in knee extension ROM. Baseline comparisons between the subgroups revealed no significant differences in VAS and TUG; however, WOMAC and ROM values differed significantly at baseline.

## 4. Discussion

The presented results indicate the preliminary evidence of treatment-associated changes following the novel double-coil rPMS approach in the treatment of patients with KOA. In addition to statistically significant changes, the achieved improvements can also be considered clinically meaningful, particularly for VAS, WOMAC, and TUG, which reached MCID responder rates exceeding 70%. These values markedly exceed those reported by Coleman et al., who examined MCID responder rates in KOA patients following a six-week self-management program. In their study, the proportion of responders did not exceed 30% for VAS and WOMAC, and reached only 46% for TUG [37]. Although differences in study design and intervention intensity preclude direct comparison, the magnitude of clinical response observed in the present cohort appears notable. In contrast, the MCID responder rate for ROM (flexion and extension combined) in our study was lower (46.7%). Exercise-based interventions remain the cornerstone of conservative KOA management and have demonstrated beneficial effects on pain, function, and oxidative stress–related pathways [12]. In this context, rPMS should not be viewed as a replacement for structured exercise therapy but rather as a potentially complementary modality that may enhance neuromuscular activation and symptom control, particularly in patients with limited tolerance to active rehabilitation. Improved functional mobility, reflected by better TUG performance, may also have biomechanical implications. Biomechanical modeling studies suggest that changes in ankle function and muscle activation patterns can modify tibiofemoral stress distribution and influence knee joint loading during dynamic activities, potentially affecting degenerative processes [38].

Interpreting the percentage improvements of individual outcome measures within the context of existing rPMS evidence remains challenging due to the limited number of comparable clinical studies in KOA populations. Keesukphan et al. investigated the immediate effects of a single session of single-coil rPMS in patients with KOA and did not demonstrate a statistically significant benefit compared with a sham group [39]. However, the small sample size and the application of only one treatment session limit the interpretability of these findings. Importantly, the study also highlights the potential contribution of placebo effects in immediate post-rPMS improvement. In contrast, Lee and Nam applied 20 sessions of single-coil rPMS in a small cohort (10 patients per group) without stratification by KL grade. Despite differences in protocol intensity and study design, the relative improvements in pain and function were broadly comparable to those observed in the present study. In their study, VAS improved by approximately 45%, compared with 50% in our cohort, and WOMAC improved by about 30%, compared with 23% in our sample. Although the smaller number of sessions in our protocol may suggest potential efficiency of the double-coil configuration, future studies with larger samples are needed to directly compare the clinical efficacy of single- and double-coil rPMS approaches.

Recently published theoretical analyses further support the potential of the double-coil rPMS setup, particularly for targeting deep structures of large joints [14]. Finite element modeling comparing both approaches demonstrated that, within depths of 3–8 cm, the double-coil configuration delivered 45–121% more magnetic energy to the knee cartilage and ligaments [15].

Articular cartilage and cruciate ligaments are typically located within this depth range, especially in individuals with increased soft tissue thickness. This may be characteristic of athletic populations with greater muscle mass as well as obese patients with increased subcutaneous adipose tissue. Both groups are at elevated risk of knee joint injury or osteoarthritis progression [40,41]. In such cases, the rapid attenuation of magnetic field intensity with distance, typical of single-coil systems, may limit effective stimulation of deeper joint structures. By contrast, the opposing placement of two coils at an adjustable angle of approximately 90° may result in partial superposition of magnetic fields and enhanced field strength at greater depths [15]. Although these assumptions are derived primarily from theoretical modeling rather than direct in vivo measurements, they offer a plausible mechanistic explanation for the clinical effects observed in this study and support further investigation of the double-coil approach in KOA.

Subgroup analysis assessing the influence of KL grade on the percentage improvement in outcome measures revealed interesting findings. Pain reduction was more pronounced in patients with KL < 3 (62.4% vs. 38.8%), whereas functional parameters such as WOMAC and ROM improved more substantially in patients with KL ≥ 3 (WOMAC: 27.0% vs. 17.5%; ROM flexion: 4.7% vs. 2.5%; ROM extension: 5.9% vs. 1.4%). Similarly, Tran et al. compared changes in VAS and WOMAC among patients with KL grades 2 and 3 and found that individuals with higher KOA grades achieved greater improvement following stromal vascular fraction treatment [42]. In contrast, Kumar and Venkatesh did not demonstrate a statistically significant difference in VAS or WOMAC between KL 2 and KL 3 patients, but they did report greater improvement in the Lequesne index following low-level laser therapy, again favoring patients with more advanced KOA [43].

The difference in pain and functional improvements across patient cohorts with varying KL grades may reflect both the nature of KOA and the underlying mechanisms of rPMS therapy. In lower KL grades, pain is likely dominated by nociceptive sources, whereas higher grades are more often associated with neuropathic pain features and central sensitization [44]. While rPMS primarily acts through peripheral mechanisms—such as reduction in muscular tension, improved local circulation, and modulation of nociceptive transmission via the spinal gate control theory—in neuropathic or centrally sensitized pain, it may exert its effects through long-term neuroplastic adaptations mediated by modulation of cortical and spinal excitability [45,46,47]. It should be emphasized that baseline WOMAC and ROM values differed significantly between subgroups, with patients in the KL ≥ 3 category presenting greater functional impairment prior to treatment. It is plausible that pain relief in patients with lower KL grades occurred during the 8-session treatment course, whereas in those with higher KL grades, achieving a similar degree of improvement might require a longer treatment protocol or extended follow-up. In the case of functional improvement measured by the WOMAC index, a perceptual component may have contributed to the results. Patients with more advanced KOA are likely to subjectively perceive the achieved change as more meaningful compared to those with milder disease, for whom baseline functional limitations are less pronounced. However, this explanation does not apply to ROM, which represents an objectively measured parameter. In this case, a more likely explanation may lie in the significantly different baseline ROM values, which provided greater potential for measurable improvement in patients with higher KL grades. Given the small sample size and the absence of a control group, these subgroup findings should be considered exploratory and hypothesis-generating.

Importantly, exploratory multivariable regression analysis adjusting for age and BMI did not identify KL grade as an independent predictor of VAS improvement. This suggests that the observed subgroup differences may reflect complex interactions between disease severity and symptom domains rather than a simple linear relationship. However, given the limited sample size, these findings should be interpreted cautiously.

Further studies incorporating larger cohorts and controlled designs are required to clarify whether KL grade independently modifies treatment response to double-coil rPMS.

### Study Limitations

The present study, given its pilot nature, has several limitations that should be acknowledged. The most significant limitation is the absence of a control group. The decision not to include a sham or control arm was based on the exploratory nature of this pilot study, which aimed to assess feasibility, safety, and preliminary clinical response under routine clinical conditions. Introducing a sham-controlled design at this stage would have required logistical restructuring beyond the scope of this feasibility-oriented investigation. Without a control or sham arm, it is not possible to attribute the observed improvements solely to the intervention, as spontaneous symptom fluctuation, regression to the mean, and placebo effects may have contributed to the results. Considering the chronic and degenerative nature of KOA, a control arm would primarily serve to distinguish the specific treatment effect of double-coil rPMS from non-specific effects and to enable comparison with established approaches (e.g., standard single-coil stimulation and/or usual care). It should be noted, however, that the present cohort consisted of patients with stable chronic symptoms, in whom spontaneous large-scale improvement across multiple clinical parameters over a short three-week period would be less likely. Baseline differences and potential confounding factors (e.g., age, BMI, physical activity level, medication use, and comorbidities) could not be fully controlled in this single-arm design and may have influenced treatment response. The number of patients included in the study was also relatively small, and the subgroup analysis assessing the influence of KL grade on percentage changes in outcome measures would require substantially larger cohorts. Given the absence of a follow-up period, the present study evaluates only the immediate post-treatment effects. These improvements may have been influenced by non-specific factors, including placebo effects, and do not allow determination of the durability of the observed benefits. Based on the proposed mechanisms of action and existing rPMS literature, a short- to mid-term persistence of effects may be anticipated; however, this assumption requires confirmation in future studies incorporating follow-up assessments of at least 3–6 months [48].

Despite these limitations, the present research provides preliminary evidence of feasibility and potential clinical benefit. To the best of our knowledge, this is the first study to investigate a double-coil rPMS approach in the treatment of KOA in a clinical cohort, providing preliminary safety and treatment-associated outcome data that can inform future trial design. The results suggest treatment-associated changes consistent with potential benefit in both early and more advanced stages of the disease and support the feasibility of delivering the protocol within a short treatment course. Such therapy may offer symptomatic relief for patients seeking to postpone surgical intervention or for those facing long waiting periods before surgery. However, these findings should be interpreted as preliminary and hypothesis-generating until confirmed in adequately powered randomized sham-controlled trials with longer follow-up.

## 5. Conclusions

This pilot study provides preliminary evidence of potential clinical benefit and feasibility of the novel double-coil rPMS approach in the treatment of patients with KOA and demonstrated treatment-associated improvements in pain, functional ability, and joint mobility. The achieved MCID responder rates exceeded 80% for VAS and TUG, exceeded 70% for WOMAC, and approached 50% for joint mobility.

Subgroup analysis suggested that disease severity may influence the pattern of response, with patients of lower KL grades experiencing greater pain reduction, whereas those with higher grades showed greater functional gains. These subgroup findings should be interpreted as exploratory.

Although these findings require validation in larger controlled trials, the initial clinical results indicate that this novel rPMS approach may represent a complementary non-invasive modality associated with short-term improvements in quality of life in patients with mild-to-moderate KOA.

## Figures and Tables

**Table 1:** Statistical summary of outcome measures obtained at baseline (Pre), after four treatment sessions (4Tx), and after completion of the treatment course (Post).

		Average ± SD/Median (IQR)	*p*	MCID Responder Rate (Pre–Post)	Effect Size (Pre–Post)
VAS	Pre	5.77 ± 1.81	F(2.58) = 44.41, *p* < 0.001	80.00%	0.605 (large)
	4Tx	3.82 ± 2.17			
	Post	3.03 ± 1.97			
	Δ	2.73 ± 1.68			
	%Δ (%)	49.85 ± 30.76			
WOMAC	Pre	62.81 ± 17.84	<0.001	73.33%	1.2 (large)
	Post	74.68 ± 16.23			
	Δ	11.87 ± 9.89			
	%Δ (%)	22.58 ± 21.73			
ROM Flexion	Pre (°)	122.5 (116.25, 130)	<0.001	46.7%	0.9 (large)
	Post (°)	130 (120, 130)			
	Δ (°)	0 (0, 10)			
	%Δ (%)	3.64 ± 4.56			
ROM Extension	Pre (°)	5 (0, 10)	0.002	46.67%	0.76 (large)
	Post (°)	0 (0, 5)			
	Δ (°)	0 (−5, 0)			
TUG	Pre (s)	11.14 (9.81, 12.75)	<0.001	83.33%	1.1 (large)
	Post (s)	9.44 (8.38, 10.42)			
	Δ (s)	1.79 (0.99, 2.79)			
	%Δ (%)	16.48 ± 9			

Data are presented as mean ± standard deviation (SD) for normally distributed variables and as median (interquartile range, IQR) for non-normally distributed variables, according to Shapiro–Wilk testing. VAS (Visual Analog Scale), WOMAC (Western Ontario and McMaster Universities Osteoarthritis Index), ROM (range of motion), and TUG (Timed Up and Go test) represent the evaluated outcome measures. VAS differences across three time points were analyzed using repeated measures ANOVA; other within-group comparisons were assessed using paired parametric or non-parametric tests as appropriate. Effect sizes are reported as partial eta squared (η^2^p) for ANOVA, Cohen’s d for parametric comparisons, and rank-biserial correlation (r) for non-parametric comparisons. MCID denotes minimal clinically important difference. Δ represents absolute change from baseline, and %Δ represents percentage change. Percentage change was not calculated for knee extension, as baseline values were often zero; therefore, absolute change in degrees (°) is reported instead. A two-sided *p*-value < 0.05 was considered statistically significant.

**Table 2:** Comparison of outcome measures between patient subgroups with knee osteoarthritis classified as Kellgren–Lawrence (KL) grade < 3 (KOA < 3) and KL grade ≥ 3 (KOA ≥ 3).

	KOA < 3 (Pre)	KOA < 3 (Δ%)	KOA ≥ 3 (Pre)	KOA ≥ 3 (Δ%)	*p*	Better in
VAS	5.29 ± 2.05	62.4 ± 30.6	6.19 ± 1.51	38.8 ± 27.3	0.54	KOA < 3
WOMAC	69.87 ± 17.53	17.5 ± 16.2	56.64 ± 16.19	27.0 ± 25.3	0.41	KOA ≥ 3
ROM Flexion (°)	130 (7.5)	2.5 ± 4.5	120 (16.25)	4.7 ± 4.5	0.13	KOA ≥ 3
ROM Extension (°)	0 (5)	1.4 ± 5.7	10 (15)	5.9 ± 8.9	0.03	KOA ≥ 3
TUG (s)	10.82 (2.88)	15.6 ± 8.7	11.84 (3.09)	17.3 ± 9.5	0.69	Comparable

Data are presented as mean ± standard deviation (SD) for normally distributed variables and as median (interquartile range, IQR) for non-normally distributed variables, according to Shapiro–Wilk testing. VAS (Visual Analog Scale), WOMAC (Western Ontario and McMaster Universities Osteoarthritis Index), ROM (range of motion), and TUG (Timed Up and Go test) represent evaluated outcomes. For all variables, positive change values represent improvement (decrease in pain or time, or increase in function or range of motion). Δ% indicates percent change (%) for VAS, WOMAC, ROM flexion, and TUG and absolute change in degrees (°) for ROM extension due to zero baseline values in most patients. Values are presented as mean ± SD. Between-group differences were analyzed using the Mann–Whitney U test. “Better in” identifies the subgroup showing greater improvement. A two-sided *p*-value < 0.05 was considered statistically significant.

## Data Availability

The data presented in this study are available from the corresponding author upon request. The data are not publicly available due to ethical and privacy restrictions.

## Abbreviations

The following abbreviations are used in this manuscript:

KOA	Knee osteoarthritis
rPMS	Repetitive peripheral magnetic stimulation
VAS	Visual analog scale
WOMAC	Western Ontario and McMaster Universities Osteoarthritis Index
TUG	Timed Up and Go
ROM	Range of motion
OA	Osteoarthritis
MCID	Minimal Clinically Important Difference
KL	Kellgren-Lawrence
BMI	Body mass index
SD	Standard deviation
IQR	Interquartile range
PRM	Physical and Rehabilitation Medicine
